# Prevalence and Predictors of Clinically Significant Depressive Symptoms Among Chinese and Malawian Children: A Cross-Cultural Comparative Cross-Sectional Study

**DOI:** 10.5539/gjhs.v7n1p59

**Published:** 2014-08-15

**Authors:** Maggie Zgambo, Fatch Welcome Kalembo, Honghong Wang, Guoping He, Sanmei Chen

**Affiliations:** 1 Department of Nursing, Xiangya Medical College, Central South University, Tongzipo Road 172, Changsha 410013, China; 2 Faculty of Health Sciences, Mzuzu University, Mzuzu, Malawi

**Keywords:** depressive symptoms, family structure, prevalence, predictors, Chinese children, Malawian children

## Abstract

**Background:**

Multicultural comparative studies have recently increased scientific knowledge base regarding the mental health of diverse populations. This cross-cultural study was cross-sectionally designed to assess differences in the prevalence and predictors of clinically significant depressive symptoms between Chinese and Malawian children.

**Methods:**

A total of 478 children (237 Chinese and 241 Malawians) were randomly recruited in the study. The participants completed a Children Depression Inventory in the dimensions of Negative Mood, Interpersonal Problems, Ineffectiveness, Anhedonia, and Negative Self- Esteem. They further provided demographic and family structure information. Data were analyzed by Student’s t-test, Chi-square test, and logistic regression.

**Results:**

The prevalence of clinically significant depressive symptoms was 16% and 12.4% for Chinese and Malawian study participants, respectively. Multivariate logistic regression analysis showed that fighting among siblings (adjusted odds ratio [aOR] = 4.1, 95% CI, 3.5–5.9), fighting among children and parents (aOR = 7.7, 95% CI, 4.6–9.8) and living with father only (aOR = 4.1, 95% CI, 3.4–6.7) were significant predictors of clinically significant depressive symptoms among Chinese study participants. On the other hand, clinically significant depressive symptoms were predicted by employment status of a mom only among Malawian study participants (aOR = 3.0, 95% CI, 2.3–5.9).

**Conclusions:**

We conclude that diverse cultures affect children’s mental health differently and this cluster of children has a noticeable amount of depressive symptoms that in the least requires further diagnosis and preventive measures.

## 1. Introduction

There is growing knowledge on different levels of depression from comparative studies which have had sufficient numbers of children from different socio-economic and racial/ethnic backgrounds. The depression prevalence and risk increase among children and the conceptual organization of the symptoms characterizing depression have been associated with different race and cultural background (Park et al., 2010; Seaton, Caldwell, Sellers, & Jackson, 2010). Prevalence rate of depression and pattern of occurrence have appeared to be different among children from diverse race and cultural backgrounds. For instance, a large study by Roberts and colleagues, an ethnically diverse sample of over 5,000 Anglo, African, Chinese, Mexican, and Native American students in Grades 6 through 8 were assessed for major depression. The results showed that Mexican American youths had higher rates of depression than other groups and their socioeconomic status was relatively lower than their counterparts (Roberts, Roberts, & Chen, 1997) argumentatively, Blazer, Kessler, McGonagle, and Swartz (1994) found the opposite of this. Additionally, a longitudinal study conducted by Chen, Rubin, and Li (1995) among primary Chinese children in Shanghai found a mean Children Depression Inventory (CDI) score of 10.72 among the study participants which compared favourably with the CDI score of 9.44 as reported in a study conducted in the West (Cole & Carpentieri, 1990).

Many cultural comparative studies conducted on the subject have focused on reporting similarities (Fichter, Xepapadakos, Quadflieg, Georgopoulou, & Fthenakis, 2004; Greenberger, Chen, Tally, & Qi, 2000) Moreover, much of what we know about comparative studies among children’s mental disorders are largely conducted and compared amongst western cultures, and/or compared among western and other cultures. Research agenda, preventive strategies and treatment approaches are often developed from this vantage summit, which may be inappropriate or unwarranted for populations in other cultures. Depressive symptoms may not be similarly expressed between different cultures. A culture’s view of depression, perception, interpretation and expression of each depression symptom (e.g. being sad) may be different across ethnic groups. For instance, in some cultures, distress in the face of multiple stressors, including discrimination, racism, and poverty, may be expected and, thus, not experienced as sadness, while in others, the same will trigger a handful of depressive symptoms (Ayalon & Young, 2003).

Moreover studies comparing ethnocultural groups have been conducted at least among adolescents in the United States of America in the manifestation of depression (Mikolajczyk, Bredehorst, Khelaifat, Maier, & Maxwell, 2007; Roberts et al., 1997; Saluja et al., 2004). The phenomenology of depression and differences in occurrence among children has been neglected. It is therefore important to enhance the scientific knowledge base regarding the mental health of ethnic minority populations, and ensuring the provision of culturally competent services which have been identified as public health priority (United States Department of Health & Human Services, 2001). Published studies have at least investigated the ethno-cultural variations of depressive symptoms among Western and Asian cultures or Western and African cultures. To the authors’ knowledge, no studies published to date have investigated the influence of sociocultural factors such as gender, age, family factors and ethnicity on manifestation of depressive symptoms among Asian and black African children. This study was therefore designed to investigate the prevalence and predictors of clinically significant depressive symptoms among Chinese and Malawian children.

## 2. Materials and Methods

### 2.1 Sample

The study was conducted in Changsha city in central China and Lilongwe city in central Malawi. The Chinese sample consisted of 237 children (123 boys and 115 girls, mean age of 10 years, SD=0.958) from three randomly selected urban primary schools in Changsha city. The participants of the study were children within the age range of 8 to 12 years, enrolled in grades 3, 4 and 5. These grades were selected because the majority of the students were in the age group of interest. All participants in China were Chinese and born in China. The Malawian sample consisted of 241 children (103 boys and 131 girls, mean age of 11 years, SD=10.57) from three randomly selected urban primary schools in Lilongwe city. The Malawian sample had children within the age group of 8 and 12, enrolled in standard 3, 4, 5 and 6. All the participants in Malawi were Malawians and born in Malawi.

### 2.2 Ethics Statement

Approval to conduct the study was obtained from the Central South University, School of Nursing Institutional Review Board. Permission was further obtained from the headmasters of the six public primary schools of concern to conduct the study. Written informed consent was also obtained from the parents of the children. All the participants were informed that they had a right to participate or withdraw from the study.

### 2.3 Procedure

Lists of students’ names in concerned grades of primary schools of interest in China and Malawi were obtained from the headmasters. Students per school were then randomly selected from the lists. The selected participants were given consent forms to take home for their parents to sign. The participants were also given questionnaires related to the parents’ level of education and employment status, for their parents to fill in. The participants brought the signed consent forms and completed questionnaires the next day. Study participation of children was voluntarily despite obtaining written informed consents from parents. Among the eligible Chinese students, 3 students did not return consent forms from parents. All Chinese and Malawian participants were uniformly asked to complete the questionnaires in classroom setting, administered by trained research assistants and teachers. All the items were uniformly read aloud to the students in all settings by the research assistants and teachers who were both present in the classrooms throughout data collection to maximize independent and confidential responses as well as offer guidance and assistance to the study participants. A total of 243 Chinese students and 246 Malawian students answered the questionnaires representing a 98.7% and 100% response rate, respectively. Six and five questionnaires from Chinese and Malawian participants respectively, were excluded from analysis because they were incomplete. This study was conducted between July and November 2012.

### 2.4 Measures and Instrumentation

#### 2.4.1 Demographic Information

The participants completed a structured questionnaire about demographic information (age, gender and religion), family structure, and family relationship. Highest educational level and employment status of parents were obtained from the parents. Family structure was assessed by asking questions on number of siblings, family members who lived with study participants in the primary home and presence of biological parents. Presence of biological parents was assessed as follows: 1) both parents were present, 2) mother only, 3) father only, 4) none. Parents of the students were asked to mention the highest level of education in the following coding: 0) no formal education, 1) primary, 2) secondary or 3) tertiary. Parents were also asked about their occupational status as follow: 1) both parents working, 2) father only working, 3) mother only working and 4) none of the caretakers were working. Parents’ relationship was assessed by asking the students how often did their parents quarrel or fight (classified as none, sometimes and many times). We further asked the students to indicate if parents and siblings or siblings themselves fought or quarrelled.

#### 2.4.2 Depression Symptoms

Children’s Depression Inventory (CDI) was used to assess depressive symptoms in Children. CDI is a validated questionnaire originally developed by M. Kovacs (1992) and has been widely used to measure depressive symptoms for children and adolescents in epidemiological studies (Cowell, Gross, McNaughton, Ailey, & Fogg, 2005; Li et al., 2007). It has five sub-scales namely; Negative Mood, Interpersonal Problems, Ineffectiveness, Anhedonia and Negative Self-Esteem. It contains 27-items with a self-rating scale per item ranging from 0 to 2. For example, do you ever feel alone? 0 = I do not feel alone; 1 = I feel alone many times; 2 = I feel alone all the time. CDI total scores vary between 0 (no depressive symptoms) and 54 (all depressive symptoms present). A 19-point cut-off was used and is the ideal threshold discriminating children at risk of depression from non-depressed children (Kovacs, 1992). Higher scores indicate severity of depression symptomatology. Participants with scores above the cut-off score are considered as having clinical significant depressive symptoms. Total score was the overall summation of points accumulated in all depressive symptoms across the five factor scales. English and Chinese versions of CDI with adequate test-retest reliability, internal consistency, and construct validity were used for Malawian and Chinese children respectively and this tool has been widely used among Chinese and African populations (Chao, Chen, Wang, Wu, & Yeh, 2003; Hong et al., 2009; Ndetei et al., 2007; Suliman et al., 2009). The CDI English version for the Malawian children was translated to Malawian local language and pretesting of the questionnaire was done among 31 pupils prior to data collection.

#### 2.4.3 Statistical Analysis

The Statistical Package for the Social Sciences (SPSS for Windows version 20) program was used for the statistical analyses. Internal consistency reliability of the CDI and its subscales was measured with Cronbach’s alpha coefficients for the Malawian translated CDI version. Descriptive statistics were used to analyze general characteristics of the study participants. Student’s t-test was used to compare means of age and CDI scores between Chinese and Malawian children. Comparison of categorical variables was carried out by using Chi-square test. Variables significant at bivariate analysis level were entered in a multistage stepwise logistic regression analysis model, and variables significant at *P* < 0.05 were retained in the final model.

## 3. Results

### 3.1 Reliability of the Malawian Translated CDI

The Cronbach’s alpha for the 27-items of the CDI, which was translated to Malawian local language (Chichewa) was 0.73. This falls within the acceptable range of between .71 and .91 as reported in previous studies (Cole & Martin, 2005; Kovacs, 1992; Wagner et al., 2003). Internal consistency reliability estimates for the five subscales of the CDI ranged from 0.56 to 0.67, with Ineffective subscale being the least reliable. These findings relate favourably to the findings of original English version of CDI in which the internal consistency for the subscales ranged from 0.59 to 0.68 (Kovacs, Goldston, & Gatsonis, 1993).

### 3.2 Depressive Symptoms

Data for 478 participants were analyzed. Table 1, shows the distribution of demographic information of both groups of participants in relation to depressive symptoms across all independent variables. Based on the CDI cut-off point of 19, 16% of Chinese children had clinically significant depressive symptoms, 34.2% of these were girls and 65.8% were boys. A proportion of 12.4% of Malawian children had clinically significant depressive symptoms, 60% were girls and 40% were boys. Almost 45% of the Chinese participants with clinically significant depressive symptoms were 10 years old while 43% Malawian children with clinically significant depressive symptoms were 12 years old. The overall mean CDI score among the Chinese study participants was 10.52 while that of Malawian participants was 10.25. The mean total score for Chinese girls was 9.52 compared to 11.46 for Chinese boys (t = 3.712, *P* > 0.05), and the total mean score for Malawian girls was 10.98 compared to 9.27 for Malawian boys (t = -1.985, *P* = 0.048). On total subscale mean scores between the groups, Chinese children had a significant higher mean score than Malawian children on negative self-esteem *(P*<0.001) whilst Malawian children scored higher on anhedonia (P < 0.05) as shown in Table 2.

**Table 1:** Chi-square test of the association between depressive symptoms and demographic data

VARIABLE	CHINESE CHILDREN			MALAWIAN CHILDREN		

	*Depressive symptoms*	*No Depressive symptoms*	*P-*value	*Depressive symptoms*	*No Depressive symptoms*	*P-value*
**Age**			.058			.195
8	0(0.0%)	15(7.5%)		2(40.0%)	3(60.0%)	
9	6(15.8%)	35(17.6%)		1(7.7%)	12(92.3%)	
10	17(44.8%)	71(35.7%)		9(18.4%)	40(81.6%)	
11	11(28.9%)	73(36.7%)		5(10.9%)	41(89.1%)	
12	4(10.5%)	5(2.5%)		13(10.2%)	115(89.8%)	
**Gender**			.054			.746
Male	25(65.8%)	97(48.7%)		12(40.0%)	91(43.1%)	
Female	13(34.2%)	102(51.3%)		18(60.0%)	120(56.1%)	
**Grade**			.168			.160
3	2(5.3%)	29(14.6%)		1(3.3%)	29(13.7%)	
4	6(15.8%)	42(21.1%)		6(20.0%)	28(13.3%)	
5	30(78.9%)	128(64.3%)		0(0.0%)	12(5.7%)	
6	0 (0.0%)	0(0.0%)		23(76.7%)	142(67.3%)	
**Religion**			.187			.339
Christianity	1(5.3%)	3(2.7%)		23(79.3%)	157(88.8%)	
Buddhism	1(5.3%)	14(12.4%)		0(0.0%)	0(0.0%)	
Islam	0 (0.0%)	0(0.0%)		6(20.7)	20(11.2%)	
Others	0(0.0%)	17(15.0%)		0(0.0%)	0(0.0%)	
None	17(89.4%)	79(69.9%)		0(0.0%)	0(0.0%)	
**Lives with**			.024			.761
Both parents	24(63.2%)	161(83.0%)		25(86.2%)	153(85.0%)	
Mom only	9(23.7%)	24(12.4%)		4(13.8%)	17(9.4%)	
Dad only	4(10.5%)	4(2.1%)		0(0.0%)	3(1.7%)	
Others	1(2.6%)	5(2.6%)		0(0.0%)	7(3.9%)	
**Number of siblings**			052			.790
1	26(68.4%)	155 (79.9%)		0(0.0%)	1(0.6%)	
2	8(21.1%)	34 (17.5%)		1(3.6%)	22(12.2%)	
3	4(10.5%)	5(2.6%)		6(21.4%)	38(21.1%)	
4	0(0.0)%	0(0.0%)		7(25.0%)	44(24.4%)	
>4	0(0.0%)	0(0.0%)		14(50.0%)	75 (41.7%)	
**Number of people in the house house**			.365			.186
2	3(7.9%)	12(6.2%)		0(0.0%)	0(0.0%)	
3	15(39.5%)	89(46.1%)		0(0.0%)	1(0.6%)	
4	9(23.7%)	49(25.4%)		0(0.0%)	18(10.0%)	
<4	11(28.9%)	43(22.3%)		29(100.0%)	161(89.4%)	
**Parents’ educational level**			.019			.032
Primary level	7(18.9%)	10(5.4%)		7(24.1%)	49(27.7%)	
Secondary level	11(29.7%)	65(35.1%)		15(51.7%)	50(28.2%)	
Tertiary level	19(51.4%)	110(59.5%)		7(24.1%)	78(44.1%)	
						
**Parents employment status**			.044			.033
Both are working	24(82.8%)	125(70.6%)		11(39.3%)	72(46.1%)	
Father only	5(17.2%)	41(23.2%)		7(25.0%)	63(40.4)	
Mother only	0(0.0%)	5(2.8%)		8(28.6%)	17(10.9%)	
Both not working	0(0.0%)	6(3.4%)		2(7.1%)	4(2.6%)	
**Family members’ relationship**			<.001			.222
No fights/quarrels	15(44.1%)	149(80.5%)		23(79.4%)	156(86.7%)	
Kids fight/quarrel	11(32.4%)	26(14.1%)		3(10.3%)	18(10.0%)	
Parents and kids fight/quarrel	8(23.5%)	10(5.4%)		3(10.3%)	6(3.3%)	
**Parents fight/quarrel**			.166			
No	13(36.1%)	98(51.0%)		25(86.2%)	137(76.1%)	.114
Sometimes	21(58.3%)	90(46.9%)		2(6.9%)	39(21.7%)	
Many times	4(5.6%)	2(2.1%)		2(6.9%)	4(2.2%)	
**Extended family**			.856			.579
No	23(62.2%)	123(63.7%)		20(69.0%)	133(73.9%)	
Yes	14(37.8%)	70(36.3%)		9(31.0%)	47(26.1%)	

**Table 2:** Mean score of CDI subscales between Chinese and Malawian children

	Chinese children	Malawian children	*P-value*

Variable	Mean /SD	Mean /SD	
Negative Mood,	2.22/2.479	2.18/1.833	0.853
Interpersonal	1.47/1.287	1.33/1.439	0.247
Ineffectiveness,	1.86/1.731	2.13/1.688	0.078
Anhedonia	2.98/1.74	3.57/2.557	0.021
Negative Self-esteem	2.03/1.88	1.13/1.313	0.000

*P-value significant at 0.05*.

### 3.3 Predicting of Clinically Significant Depressive Symptoms for Malawian Children

This study was also seeking to find whether clinically significant depressive symptoms could be predicted by the following factors among both groups: age, religion, number of siblings, presence of both parents, fighting of parents, fighting of family members, parents’ level of education and extended family. Depressive symptoms were regarded as dependent variable and the rest were considered as independent variables. Variables that were significant at bivariate analysis level were entered in logistic regression model. Only two variables (parents’ educational level and parents occupational status) were significant (P < 0.05) at bivariate level and were included in logistic regression analysis for Malawian children. After adjusting for parental level of education, it was found that children from homes where only a mother was working, were three times more likely to have clinically significant depressive symptoms than those who came from homes where both parents were working (aOR = 3.0, 95% CI, 2.3–5.9) (see Table 3).

**Table 3:** Multivariate analysis of factors associated with depressive symptoms among Malawian children

Variable	Depressive symptoms	OR	95% CI	*P*-value	aOR	95% CI	*P*-value
**Parents’ educational level**							
Primary	7(12.5%)	1.0					
Secondary	15(23.1%)	2.1	.7-5.6	.138			
Tertiary	7(8.2%)	.6	.2-1.9	.410			
**Parents’ employment status**							
Both work	11(13.3%)	1.0					
Father only	7(10.0%)	.7	.3-2.0	.535			
Mom only	8(32.0%)	3.3	2.5-6.4	.036	3.0	2.3-5.9	.046
Both are unemployed	2(33.3%)	3.3	.5-20.0	.200			

***OR**: Odds ratio, **aOR**: Adjusted odds ratio, **95% CI**: 95 percent confidence interval, **1.0**: reference group, (**aOR** adjusted for all variables in the table)*.

### 3.4 Predicting of Clinically Significant Depressive Symptoms for Chinese Children

Using the same procedure explained above, four variables (family relationship, parents’ level of education, gender and parent/s living with the child) were found significant at bivariate analysis level for Chinese children and were included in logistic regression analysis. After adjusting for family relationship, parental level of education, and parent living with the child, it was revealed that fighting among siblings (aOR = 4.1, 95% CI, 3.5-5.9), fighting among children and parents (aOR = 7.7, 95% CI, 4.6–9.8) and living with father only (aOR = 4.1, 95% CI, 3.4–6.7) were shown to be significant predictors of clinically significant depressive symptoms for Chinese children (see Table 4).

**Table 4:** Multivariate analysis of factors associated with depressive symptoms amongChinese children

Variable	Depressive symptoms	OR	95%CI	*P*-value	aOR	95%CI	*P*-value
**Family relationship**							
Love each other	15(9.1%)	1.0			1.0		
Kids fight	11(29.7%)	4.2	3.7- 6.2	<.001	4.1	3.5 - 5.9	.001
Kids and parents fight	8(44.4%)	7.9	4.7 -10.2	<.001	7.7	4.6 - 9.8	.001
**Parents’ level of education**							
Primary	7(41.2%)	1.0					
Secondary	11(14.5%)	.2	.1-.8	.016	.3	.1- .8	.024
Tertiary	19(14.7%)	.2	.1-.7	.011	.3	.1-.8	.019
**Parent living with child**							
Dad and mum	24(13%)	1.0			1.0		
Mum only	9(27.3%)	2.5	1.1- 6.1	.039	NS		
Dad only	4(50%)	5.1	4.3 - 7.2	.010	4.1	3.4 - 6.7	.032

***OR:** Odds ratio, **aOR**: Adjusted odds ratio, 95% **CI**: 95 percent confidence interval, **1.0**: reference group, **NS**: not significant, (**aOR** adjusted for all variables in the table)*.

## 4. Discussion

Based on the CDI cut-off point of 19, Chinese participants had a higher rate of clinically significant depressive symptoms (16%), compared to Malawian participants (12.4%). A similar a study found high levels of depressive symptoms among Chinese adolescents as compared to South Africans (Steptoe, Tsuda, Tanaka, & Wardle, 2007). Other authors have attributed high levels of depressive symptoms among Chinese children to isolation and anxiety (Choi, 2002), and lack of encouragement for self-esteem among the Chinese culture (Kanagawa, Cross, & Markus, 2001; Kitayama, Markus, Matsumoto, & Norasakkunkit, 1997; Spencer-Rodgers, Peng, Wang, & Hou, 2004). This is reinforced in our study, where Chinese children had a higher mean score than Malawian children on negative self-esteem (P < 0.001). It should also be noted that most of the Chinese participants in this study were coming from families where they were the only children unlike their Malawian counterparts most of whom had more than four siblings. The one child per family policy employed by Chinese government might have profound effect on this. A study which compared the psychosocial outcomes between Chinese children without siblings and those with siblings found that girls who were the only children in their families had higher scores of depressive symptoms than those with siblings (Tseng et al., 1988). It may be argued that the higher number of siblings in the family among the Malawian sample provided an environment where the siblings interacted and provided support to each other to counteract the effects of stress.

Of the 16% of the Chinese children who had clinically significant depressive symptoms, 34.2% were girls (mean CDI score 9.52, SD 8.56) and 65.8% were boys, indicating that boys had relatively more depressive symptoms than girls. It is argued that in Chinese culture, parents and communities have greater expectations from boys which put much pressure on them (Shang et al., 2010). Unlike Malawian children, of the 12.4% who had clinically significant depressive symptoms, 60% were girls and 40% were boys. This is consistent with findings from a study done in Kenya (Khasakhala, Ndetei, Mutiso, Mbwayo, & Mathai, 2012), where prevalence rate of depressive symptoms among girls was 31% compared to 22.6% among boys. It is evident that girls carry more pre-existing risk factors for depression that combine with greater biological and social challenges during adolescence (Dong, Wang, & Ollendick, 2002; Wang, He, Fang, & Li, 2011).

It should be noted that the pattern of CDI scores increased with age in this study for the Chinese children (P<0.05), a similar study reported similar trend, whereby older children reported higher rates of depressive symptoms than younger children (Tepper et al., 2008). This may be explained in relation to the highly competitive style of Chinese education system as reported by X. Liu et al. (2000), school hours in China, are long and homework loads are heavy, in addition, the school authorities and parents emphasize the need for students to score high grades in their studies and this put a lot pressure on children. The pressure for academic performance increases with age, resulting to high scores of depressive symptoms. A part from this, the physical and psychological changes and challenges that come with increase in age may also result in increased depressive symptoms. Unlike the Malawian sample, there was no significant relationship between increased in age and depressive symptoms just like findings byAdewuya and Ologun (2006), who found no significant relationship between increase in age and depressive symptoms. The prevalence of clinically significant depressive symptoms in both groups indicates that depressive symptoms take root at a young age. Since depressive symptoms has a tendency of relapsing in adulthood (Reinherz, Paradis, Giaconia, Stashwick, & Fitzmaurice, 2003), it is beneficial to avoid long term capitulation by stage-managing preventive measures of clinically significant symptoms focusing on this age group in both countries. This will prevent depression and outcomes that stem from it like suicide and poor academic performance.

It was found that Chinese children from families where kids fought or they fought with parents, lived with father only were more likely to have clinically significant depressive symptoms than their counterparts. These findings are similar to earlier studies findings done in China and elsewhere (Kader Maideen, Mohd Sidik, Rampal, & Mukhtar, 2014; Z. Liu, Li, & Ge, 2009; Stoep, Weiss, Kuo, Cheney, & Cohen, 2003; Sund, Larsson, & Wichstrom, 2003), where poor relationship between family members and single parenthood were associated with high depressive symptoms. Since apart from school, children spend much of their time at home, poor relationship between family members provide a hostile and stressful environment for children which can result to depressive symptoms. The explanation behind the association between living with father only and depressive symptoms can be related to Chinese culture, in which the mother often attaches more strongly with her children than the father does (Choi, 2002), her absence deprives the children emotional support which is critical to prevention of depression. Like findings of one study (Hong et al., 2009), where children whose parents had reached secondary and tertiary education were less likely to have clinically significant depressive symptoms than those whose parents reached primary school level of education, Chinese participants whose parents had higher educational level were less likely to have depressive symptoms than those whose parents had lower educational status. High educational level is associated with good employment and high income, which can help parents to provide basic necessities to their children thereby enabling them to leave a less stressful life. Malawian children who came from homes where only a mother was employed were three times more likely to have clinically significant depressive symptoms than those who came from homes where both parents were employed. Culturally fathers are heads of the families in Malawi and are expected to support their families. Father’s unemployment status might appear as passive and helpless role model to their children and at the same time the unemployed fathers may have more somatic and psychiatric worries or illnesses than working fathers which may affect the emotional wellbeing of their children.

### 4.1 Strengths and Limitation of the Study

The major strength of this study is that it is one of the few studies that have investigated the differences in prevalence and predicators of depressive symptoms between Malawian and Chinese children. The study underscores the importance of taking into consideration ethnocultural variation in the prevention, diagnosis and treatment of depressive disorders in children. Furthermore, the study used a standard instrument with acceptable internal consistency to measure the differences in depressive symptoms among the two samples, this ensured provision of meaningful results. The study’s major limitation is the small sample size, it can neither be generalized to all children in these countries nor globally. The cross-sectional nature of the study limits its causal relationship between independent and dependent variables. This highlights the need for a longitudinal study to establish the causal direction between these variables. It should also be noted that the CDI does not provide a definite diagnosis of depression hence the results of the study should be interpreted with caution. Nevertheless, despite the CDI not yielding definite diagnosis of depression, it still identifies children with clinically significant depressive symptoms that need further diagnosis and management. Future studies should aim at finding the differences in diagnosis of depression between the two cultures using appropriate instruments.

### 4.2 Conclusion

Although the scores do not denote a clinical diagnosis of depression, this cluster of children has a noticeable amount of clinically significant depressive symptoms that in the least requires further diagnostics. Depressive symptoms are associated with poor family relationship, level of parents’ education and living with a father only among Chinese children, while for Malawian children, parents’ unemployment status was significantly associated with depressive symptoms. The study implicates unawareness among parents and teachers on depressive symptoms and their associated dangers. Programs that can assist family members and teachers to detect depressive symptoms and instigate seeking assistance from appropriate mental health service providers for cure and prevention are recommended in both places.

## References

[ref1] Adewuya A. O, Ologun Y. A (2006). Factors associated with depressive symptoms in Nigerian adolescents. J Adolesc Health.

[ref2] Ayalon L, Young M. A (2003). A comparison of depressive symptoms in African Americans and Caucasian Americans. Journal of Cross-Cultural Psychology.

[ref3] Blazer D. G, Kessler R. C, McGonagle K. A, Swartz M. S (1994). The prevalence and distribution of major depression in a national community sample:the National Comorbidity Survey. Am J Psychiatry.

[ref4] Chao C. C, Chen S. H, Wang C. Y, Wu Y. C, Yeh C. H (2003). Psychosocial adjustment among pediatric cancer patients and their parents. Psychiatry Clin Neurosci.

[ref5] Chen X, Rubin K. H, Li B. S (1995). Depressed mood in Chinese children:Relations with school performance and family environment. J Consult Clin Psychol.

[ref6] Choi H (2002). Understanding adolescent depression in ethnocultural context. Advances in Nursing Science.

[ref7] Cole D. A, Carpentieri S (1990). Social status and the comorbidity of child depression and conduct disorder. J Consult Clin Psychol.

[ref8] Cole D. A, Martin N. C (2005). The longitudinal structure of the Children’s Depression Inventory:Testing a latent trait-state model. Psychological Assessment.

[ref9] Cowell J. M, Gross D, McNaughton D, Ailey S, Fogg L (2005). Depression and suicidal ideation among Mexican American school-aged children. Res Theory Nurs Pract.

[ref10] Dong Q, Wang Y, Ollendick T. H (2002). Consequences of divorce on the adjustment of children in China. J Clin Child Adolesc Psychol.

[ref11] Fichter M. M, Xepapadakos F, Quadflieg N, Georgopoulou E, Fthenakis W. E (2004). A comparative study of psychopathology in Greek adolescents in Germany and in Greece in 1980 and 1998-18 years apart. Eur Arch Psychiatry Clin Neurosci.

[ref12] Greenberger E, Chen C, Tally S. R, Qi D (2000). Family, peer, and individual correlates of depressive symptomatology among U.S. and Chinese adolescents. J Consult Clin Psychol.

[ref13] Hong X, Li J, Xu F, Tse L. A, Liang Y, Wang Z, Griffiths S (2009). Physical activity inversely associated with the presence of depression among urban adolescents in regional China. BMC Public Health.

[ref14] Kader Maideen S. F, Mohd Sidik S, Rampal L, Mukhtar F (2014). Prevalence, Associated Factors and Predictors of Depression among Adults in the Community of Selangor, Malaysia. PLoS ONE.

[ref15] Kanagawa C, Cross S. E, Markus H. R (2001). “Who am I?”- The cultural psychology of the conceptual self. Personality and Social Psychology Bulletin.

[ref16] Khasakhala L. I, Ndetei D. M, Mutiso V, Mbwayo A. W, Mathai M (2012). The prevalence of depressive symptoms among adolescents in Nairobi public secondary schools:association with perceived maladaptive parental behaviour. African Journal of Psychiatry.

[ref17] Kitayama S, Markus H. R, Matsumoto H, Norasakkunkit V (1997). Individual and collective processes in the construction of the self:self-enhancement in the United States and self-criticism in Japan. J Pers Soc Psychol.

[ref18] Kovacs M (1992). Children’s Depression Inventory.

[ref19] Kovacs M, Goldston D, Gatsonis C (1993). Suicidal Behaviors and Childhood-Onset Depressive-Disorders - a Longitudinal Investigation. J Am Acad Child Adolesc Psychiatry.

[ref20] Li Y. P, Ma G. S, Schouten E. G, Hu X. Q, Cui Z. H, Wang D, Kok F. J (2007). Report on childhood obesity in China (5) body weight, body dissatisfaction, and depression symptoms of Chinese children aged 9-10 years. Biomed Environ Sci.

[ref21] Liu X, Kurita H, Uchiyama M, Okawa M, Liu L, Dengdai M (2000). Life events, locus of control, and behavioral problems among Chinese adolescents. Journal of Clinical Psychology.

[ref22] Liu Z, Li X, Ge X (2009). Left too early:the effects of age at separation from parents on Chinese rural children’s symptoms of anxiety and depression. Am J Public Health.

[ref23] Mikolajczyk R, Bredehorst M, Khelaifat N, Maier C, Maxwell A (2007). Correlates of depressive symptoms among Latino and Non-Latino White adolescents:Findings from the 2003 California Health Interview Survey. BMC Public Health.

[ref24] Ndetei D. M, Khasakhala L, Seedat S, Syanda J, Ongecha-Owuo F. A, Kokonya D. A, Mutiso V. A (2007). Psychometric properties of a multidimensional anxiety scale for children (MASC) amongst Nairobi public secondary school children in Kenya. Journal of Child and Adolescent Mental Health.

[ref25] Park M. H, Kim T. S, Yim H. W, Jeong S. H, Lee C, Lee C. U, Jun T. Y (2010). Clinical characteristics of depressed patients with a history of suicide attempts:results from the CRESCEND study in South Korea. J Nerv Ment Dis.

[ref26] Reinherz H. Z, Paradis A. D, Giaconia R. M, Stashwick C. K, Fitzmaurice G (2003). Childhood and adolescent predictors of major depression in the transition to adulthood. Am J Psychiatry.

[ref27] Roberts R. E, Roberts C. R, Chen Y. R (1997). Ethnocultural differences in prevalence of adolescent depression. Am J Community Psychol.

[ref28] Saluja G, Iachan R, Scheidt P. C, Overpeck M. D, Sun W, Giedd J. N (2004). Prevalence of and risk factors for depressive symptoms among young adolescents. Arch Pediatr Adolesc Med.

[ref29] Seaton E. K, Caldwell C. H, Sellers R. M, Jackson J. S (2010). An intersectional approach for understanding perceived discrimination and psychological well-being among African American and Caribbean Black youth. Dev Psychol.

[ref30] Shang X. W, Wang D, Wang J. P, Hu X. Q, Du S. M, Li Y. P (2010). Prevalence and socioeconomic status correlation of depressive symptoms among children living in urban Beijing. North American Journal of Medicine and Science.

[ref31] Spencer-Rodgers J, Peng K, Wang L, Hou Y (2004). Dialectical self-esteem and East-West differences in psychological well-being. Pers Soc Psychol Bull.

[ref32] Steptoe A, Tsuda A, Tanaka Y, Wardle J (2007). Depressive symptoms, socio-economic background, sense of control, and cultural factors in university students from 23 countries. Int J Behav Med.

[ref33] Stoep A. V, Weiss N. S, Kuo E. S, Cheney D, Cohen P (2003). What proportion of failure to complete secondary school in the US population is attributable to adolescent psychiatric disorder?. J Behav Health Serv Res.

[ref34] Suliman S, Mkabile S. G, Fincham D. S, Ahmed R, Stein D. J, Seedat S (2009). Cumulative effect of multiple trauma on symptoms of posttraumatic stress disorder, anxiety, and depression in adolescents. Comprehensive Psychiatry.

[ref35] Sund A. M, Larsson B, Wichstrom L (2003). Psychosocial correlates of depressive symptoms among 12-14-year-old Norwegian adolescents. J Child Psychol Psychiatry.

[ref36] Tepper P, Liu X, Guo C, Zhai J, Liu T, Li C (2008). Depressive symptoms in Chinese children and adolescents:parent, teacher, and self reports. J Affect Disord.

[ref37] Tseng W. S, Kuotai T, Hsu J, Chiu J. H, Yu L, Kameoka V (1988). Family planning and child mental health in China:the Nanjing Survey. Am J Psychiatry.

[ref38] United States Department of Health & Human Services (2001). Mental Health:culture,race, and ethenicity-A supplement to mental Health:A report of the Surgeon General, US department of Health and Human services, public health service.

[ref39] Wagner J. L, Chaney J. M, Hommel K. A, Page M. C, Mullins L. L, White M. M, Jarvis J. N (2003). The Influence of Parental Distress on Child Depressive Symptoms in Juvenile Rheumatic Diseases:The Moderating Effect of Illness Intrusiveness. J Pediatr Psychol.

[ref40] Wang Y. J, He B. Y, Fang L. H, Li H. J (2011). [Preliminary study on the health status among the “left-behind” children in the Xiantao rural area of Hubei Province]. Zhongguo Dang Dai Er Ke Za Zhi.

